# High Incidence of Japanese Encephalitis, Southern China

**DOI:** 10.3201/eid1904.120137

**Published:** 2013-04

**Authors:** Yun Feng, Shihong Fu, Hailin Zhang, Lyle R. Petersen, Baosen Zhang, Xiaoyan Gao, Weihong Yang, Yuzhen Zhang, Baoqing Dao, Kunhong Li, Na Li, Zhengliu Yin, Yonghua Liu, Roger Nasci, Huanyu Wang, Guodong Liang

**Affiliations:** Yunnan Institute of Endemic Disease Control and Prevention, Dali, People’s Republic of China. (Y. Feng, H. Zhang, W. Yang, Y. Zhang);; National Institute for Viral Disease Control and Prevention of the Chinese Center for Disease Control and Prevention, Beijing, China (S. Fu, X. Gao, H. Wang, G. Liang);; Centers for Disease Control and Prevention, Fort Collins, Colorado, USA (L.R. Petersen, R. Nasci);; Dehong Prefecture Center for Disease Control and Prevention, Mangshi, China (B. Zhang, B. Dao, K. Li, N Li);; Ruili Center for Disease Control and Prevention, Ruili, China (Z. Yin, Y. Liu)

**Keywords:** Japanese encephalitis, viral encephalitis, mainland China, Dehong Prefecture, viruses, Japanese encephalitis virus, People’s Republic of China, vector-borne infections

**To the Editor:** Japanese encephalitis virus (JEV) remains a major source of illness and death in Asia (*1*). An estimated 67,900 cases occur each year in Asia; ≈33,900 cases—half the cases in the world—probably occur in the People’s Republic of China (*2*). However, because reporting is incomplete in most countries where JE incidence is high, these estimates are based on scarce data. In China, a study conducted during 2006–2007 in sentinel hospitals in 1 prefecture each in Shandong, Hubei, Guangxi, and Hebei Provinces (all in the eastern half of China) found that 9.2% of patients with acute meningitis and encephalitis had JEV; adjusted incidence for each prefecture was 0.08–1.58 cases per 100,000 population (*3*). Incidence in these 4 prefectures is lower than that among children <14 years of age in JE-endemic countries, where estimated incidence is 5.4 cases per 100,000 population (*2*). To assess the need for strengthening existing JE surveillance and vaccination programs, we conducted a population-based study of JE incidence in 1 area of southern China.

Dehong is a prefecture in western Yunnan Province, which borders Myanmar. The JE-susceptible population of Dehong Prefecture (residents <15 years of age) is 211,337. The 2 principal cities of Dehong Prefecture—Mangshi and Ruili—are busy commercial centers surrounded by areas of extensive rice cultivation. The mosquito vector of JEV, *Culex tritaeniorhynchus*, is predominant during summer (*4*). During 1988–2007, JEV vaccination was available only at certain clinics and only for a fee; however, since 2008, vaccination with the live, attenuated SA 14–14–2 JEV vaccine (Chengdu Institute of Biologic Products, Chengdu, China) has been included in the national Expanded Program on Immunization at no charge. The recommendation for children is vaccination at 8 months and 2 years of age (*5**,**6*).

To estimate JE incidence in Dehong Prefecture during January 1–December 31, 2010, we conducted an anonymous, unlinked study of all cases of encephalitis at the only 2 major children’s hospitals in the region, Dehong Prefecture Hospital in Mangshi and Ruili City Hospital in Ruili. All eligible patients admitted to these hospitals were included in the study. Inclusion criteria were age <15 years, residency in Dehong Prefecture, clinical diagnosis of encephalitis, lumbar puncture performed (routine for encephalitis patients at these 2 hospitals), and cerebrospinal fluid (CSF) pleocytosis. After routine testing was completed, leftover CSF and serum samples were stored at −70°C until further testing, which was all conducted at the Chinese Center for Disease Control and Prevention in Beijing. All CSF specimens were tested by viral culture in C6/36 and BHK-21 cells (*7*) and tested for antibodies against JEV (*3**,**4*). Serum samples were tested for antibodies against JE virus, mumps virus, echoviruses, and coxsackieviruses (*3**,**4**,**7*). A case of JE was defined as illness in a person with IgM against JEV in CSF or serum. Clinical information was collected by using a standardized chart abstraction form. Linkages to personal identifiers were destroyed.

A total of 189 eligible patients were enrolled, 150 from Mangshi and 39 from Ruili. Of these, 110 (58%) were male and 78 (41%) were <4 years of age. Enrollment peaked during summer (Figure). All patients were hospitalized within 6 days after symptom onset. A total of 22 (12%) patients were classified as having JE on the basis of IgM, in CSF for 21 and in serum for 1. Illness onset occurred during May–November (Figure); overall incidence was 10.4 cases per 100,000 children <15 years of age. Among these 22 children with JE, 11 were male; 20 were from rural areas; 14 were from Mangshi and 8 were from Ruili; and 5 were 0–1 years, 6 were 2–4 years, and 11 were 5–13 years of age. JEV vaccination history was infrequently recorded in the medical charts; however, JEV was more likely to be the cause of encephalitis among children who received no vaccination (22%, 6/27) than among those with unknown vaccination history (10%, 15/157). Of 5 vaccinated children, 1 had JE; however, verification of this child’s vaccination was not possible. Among 71 children who had no evidence of JE but for whom serum samples were available for testing, 5 had antibodies against mumps virus, 8 against echoviruses, and 5 against coxsackieviruses. Viral cultures of CSF from all 189 children were negative.

**Figure:**
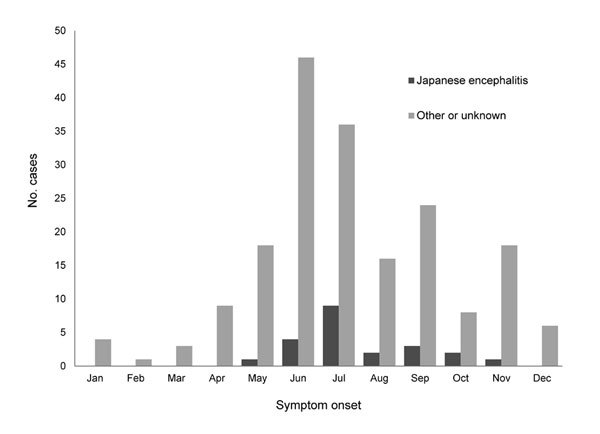
Number of children with encephalitis at 2 hospitals, by etiology and month of symptom onset, Dehong Prefecture, People’s Republic of China, 2010.

Our finding of 10.4 JE cases per 100,000 children <15 years of age in Dehong Prefecture is higher than the estimated incidence of 5.4 cases per 100,000 population among children <14 years of age in JE-endemic countries (*2*). Nevertheless, the true JE population incidence for Dehong Prefecture might be underestimated if some children received no medical care or were admitted to other hospitals. Adults were not studied; however, ≈90% of JE cases in China are reported among children <15 years of age (*5**,**6**).* Unfortunately, accurate age-adjusted JE vaccination coverage data for Dehong Prefecture are not available. Although vaccination programs have markedly lowered JE incidence in China in recent years (*5**,**6*), the finding of continuing high JE incidence in Dehong Prefecture warrants further attention.
